# A qualitative exploration of people living with idiopathic pulmonary fibrosis experience of a virtual pulmonary rehabilitation programme

**DOI:** 10.1186/s12890-022-02221-6

**Published:** 2022-11-28

**Authors:** Orlagh O’Shea, Grainne Murphy, Luke Forde, Katherine M. A. O’Reilly

**Affiliations:** 1 School of Physiotherapy, Royal College of Surgeons in Ireland, Dublin, Ireland; 2 Mater Misericordiae University Hospital, Dublin, Ireland; 3 Wexford General Hospital, Wexford, Ireland

**Keywords:** Pulmonary rehabilitation, Virtual Pulmonary Rehabilitation, Idiopathic pulmonary, Fibrosis, Qualitative

## Abstract

**Background:**

Pulmonary rehabilitation (PR) is recommended in the treatment of people with idiopathic pulmonary fibrosis (IPF). Little is known about the experiences of people with IPF of PR. Due to Covid-19 there has been a rapid shift of PR services to remote/virtual delivery.

**Objective:**

To explore people living with IPFs experience of a virtual PR (VPR) programme.

**Methods:**

All patients with a diagnosis of IPF in a stable phase of the disease were invited to participate in virtual PR: a 10 week exercise programme delivered twice-weekly for one hour. One-to-one semi- structured interviews were conducted within one week following the programme. All interviews were recorded, transcribed and analysed using Braun and Clarke thematic analysis by two independent assessors.

**Results:**

*N*=13 participants took part in the semi-structured interviews, mean (standard deviation (SD)) age 69.5(10.4) years; 7M:6F. Mean (SD) FEV_1_ 2.6(0.3)L, FVC 2.9(0.4)L. Four key themes were identified: 1) The impact of VPR on health and outlook, (2) The reality of VPR, (3) Being active after VPR and (4) Living with IPF during the COVID-19 Pandemic. Participants reported high levels of enjoyment and engagement with the programme regardless of the health benefits experienced. Most participants expressed a desire for a longer programme. Participants expressed different levels of maintenance with exercise since finishing the programme, specific motivators and strategies for maintenance included lung transplant, the maintenance of benefits from the programme and social support. COVID-19 and the restrictions imposed had some negative impacts on some participants lives, engaging with PR helped overcome some of these.

**Conclusion:**

Despite the progressive nature of IPF, all participants expressed high levels of enjoyment with the programme. Future research should explore strategies for maintenance post PR and the optimum duration of PR for people with IPF.

**Supplementary Information:**

The online version contains supplementary material available at 10.1186/s12890-022-02221-6.

## Background

Idiopathic pulmonary fibrosis (IPF) is a chronic and progressive lung disease of unknown aetiology [1]. IPF results in severe morbidity with shortness of breath and impaired quality of life due to worsening lung function [2]. IPF has a very poor prognosis with a median survival of 3–5 years [3]. Encouragingly a growing portfolio of treatment (anti-fibrotic medications) options for IPF are now available that slow the rate of disease progression and may have modest mortality benefits [3]. However these medicines are associated with some side effects and tolerability issues [4]. Lung transplantation is currently the only method to significantly improve both symptoms and survival time in people with IPF [5, 6]. According to the International Society for Heart and Lung Transplantation five year survival post lung transplant is 59% [7]. However, due to the limited availability of donor organs and the risk of chronic allograft rejection, only a few patients ever receive a lung transplant [8]. Currently, mainstay management of IPF is to manage symptoms, improve health status and preserve lung function [9].

Pulmonary rehabilitation (PR) is a non-pharmacological treatment recommended for people with IPF both nationally and internationally [10, 11]. PR is defined as a comprehensive intervention based on a thorough patient assessment followed by patient-tailored therapies. These include, but are not limited to, exercise training, education, and behavior change, designed to improve the physical and psychological condition of people with chronic respiratory disease (CRD) and to promote the long-term adherence to health-enhancing behaviors [11]. A recent systematic review and meta-analysis has demonstrated that PR can increase exercise capacity and quality of life in people with IPF [12]. However, not all patients with IPF are suitable candidates for or wish to participate in these programs [13]. Reasons for non-participation include shielding owing to risk of infection, long travelling distance to rehabilitation facilities and severely compromised mobility [14]. Therefore, tele-rehabilitation may offer an alternative for patients with IPF to ensure participation in rehabilitation programs. The COVID-19 pandemic highlighted the increased need for the delivery of virtual health care, particularly for vulnerable populations including those with IPF [15] as they were advised to cocoon to shield from infection [16]. At the beginning of the COVID-19 pandemic, a closure of centre based programs was observed globally and many programs attempted a rapid transition to home-based or telehealth models [17]. A Cochrane review reported that for people with CRDs, virtual PR (VPR) achieves outcomes similar to those of traditional centre-based PR, with no safety issues identified [18]. However this review largely pertained to research in people with COPD [18], with limited research and guidance for other CRDs including IPF.

While quantitative data gives us information on the effectiveness of PR with regard to important clinical markers, it does not tell us whether the participants actually enjoyed or liked the intervention, a recognised predictor of maintenance [19]. Furthermore, qualitative research explores the individual’s lived experiences, which can disclose subtle details and meanings not identified using quantitative methods alone [20]. The individuals’ experience of PR is valued and has been extensively researched in other respiratory populations [20]. However, research exploring people with IPF experience of PR, delivered in person or virtual is limited and to our knowledge has not yet been explored. The aim of this study was to explore people living with IPFs experience of virtual PR (VPR).

## Methods

We employed a phenomenological qualitative research design [21], consisting of individual semi-structured interviews with participants who had recently finished a VPR programme. This study was part of a wider mixed methods feasibility study which also explored the impact of VPR on physical activity levels in people with IPF, we aimed to recruit 30 participants to this trial and used the principles of data saturation to determine the qualitative sample. Here we report only the qualitative data. The Consolidated criteria for reporting qualitative research (COREQ) criteria were used to report the method and results [22]. This project was approved by the Mater Misericordiae University Hospital, Research Ethics Committee. Institutional review board reference: 1/378/2111. Written informed consent was obtained from all participants.

All methods were carried out in accordance with relevant guidelines and regulations, as per the Irish Health Service guidelines for PR [23]. All research processes were carried in accordance with local ethical guidelines.

### Participants and Recruitment

This study employed a convenience sampling frame. All individuals with IPF who were registered patients of the Mater Misericordiae University Hospital who were referred to PR were screened for eligibility by GM. Those meeting the inclusion criteria for PR: functionally limited by breathlessness and in stable phase of IPF were invited to participate by GM during a telephone consultation. A stable phase of IPF was defined as patients who do not have rapidly escalating symptoms or rapidly increasing oxygen needs. Written informed consent was obtained by post. Patients referred for palliative care were excluded from the programme as patients currently under palliative care have access to a dedicated PR programme run within the hospice and support from hospice based physiotherapy. Hence, they attend a different programme geared towards patients with more advanced disease. Those wishing to participate in the VPR but not the research aspect were not excluded from the programme, i.e. individuals could engage in the VPR but were not compelled to participate in qualitative interviews.

### Intervention

All participants underwent a 10 week VPR programme. There was no in-person PR being delivered at this time due to the COVID-19 pandemic. The VPR was delivered by a senior physiotherapist (GM) with 27 years as a senior respiratory physiotherapist. The programme was specifically designed for people with interstitial lung diseases (ILD) including IPF. The programme consisted of twice-weekly, one hour exercise classes for 10 weeks. The programme was delivered via the Salaso platform (Salaso Health Solutions, Ireland). Salaso is a video conferencing platform similar to Zoom. A full description of the intervention is available in the e-supplement.

### Data collection

All interviews were conducted via telephone by OOS due to COVID-19 restrictions. The intervention was delivered September 2020- April 2022. Data were collected between March 2021 and May 2022, COVID-19 restrictions were not lifted in Ireland until January 2022, and only one further interview took place after this, which was conducted by telephone for uniformity. Further information on COVID-19 restrictions in Ireland can be found in the e-supplement. Interviews were audio recorded and transcribed. A topic guide was developed. Following the first two interview OOS and LF met to discuss the topic guide, a further question was added to explore the impact of COVID-19 on all aspects of lifestyle. This question was added to contextualise the wider impact of VPR on individuals social and physical functioning. The finalised topic guide is available in Table 1.

**Table 1:** Semi-structured interview guide

1. How do you feel the pulmonary rehabilitation programme has affected your health? (prompts: physical health, mental health, notice any differences between before you started the programme and now) 2. What impact has COVID-19 had on your life? 3. Do you think you have a good understanding of the benefits of exercise/physical activity for someone with your condition? 4. How satisfied were you with the pulmonary rehabilitation programme? (prompts: duration, tailored to your needs, timing, frequency, technology) 5. What suggestions if any, would you give to improve the pulmonary rehabilitation programme? 6. How easy did you find it to adhere to pulmonary rehabilitation? 7. How confident are you that you could continue to exercise or do physical activity on your own now that the programme has finished? (prompts: what is helping you, what are your challenges, probe reasons for level of confidence) 8. Would you recommend this pulmonary rehabilitation programme to anyone else who has IPF? 9. Is there anything else that you would like to add regarding your experiences of taking part in the study?	

Data collection continued until the data set was deemed to be approaching thematic saturation [24]. Thematic saturation was defined as evidence of rich data with breadth and depth in relation to the study objectives, with replication of data across several participants [25]. One further interview was then conducted to confirm that saturation had been reached. To determine potential thematic saturation, we conducted a preliminary analysis of the transcripts.

Demographic data was collected from participants’ medical charts, including gender, age and lung function.

### Data analysis

All participant data was pseudo-anonymized for analysis. The data were analysed using Braun and Clarke thematic analysis [26]. The five stages of Braun and Clarke are detailed in the e-supplement.

OOS and LF coded the transcripts independently using Microsoft Word. The authors then met frequently and achieved consensus about codes through discussion, the authors kept notes of these meetings. In the third phase, OOS, LF and KOR met to elevate and combine key concepts and to identify major themes. In the final phase, OOS, LF and KOR met to examine the relationships between major themes to examine how they related to each other and to accurately represent the participants’ experiences of VPR; the themes were defined and named.

Quantitative data was entered into Microsoft Excel 2016 and analysed using descriptive statistics.

### Reflexivity

The authors have expertise in the management of patients with IPF and exercise. Author (OOS) is a lecturer in physiotherapy with experience in qualitative research. OOS has undergone qualitative research training during her PhD [27] and as postdoctoral researcher [28] and as an independent researcher exploring simulation-based learning in physiotherapy practice education. LF is respiratory specialist registrar. KOR is a consultant respiratory physician with a special interest in ILD. GM is a senior physiotherapist who delivered the VPR but was not involved in the analysis of the results to prevent any bias.

## Results

**Fig. 1:**
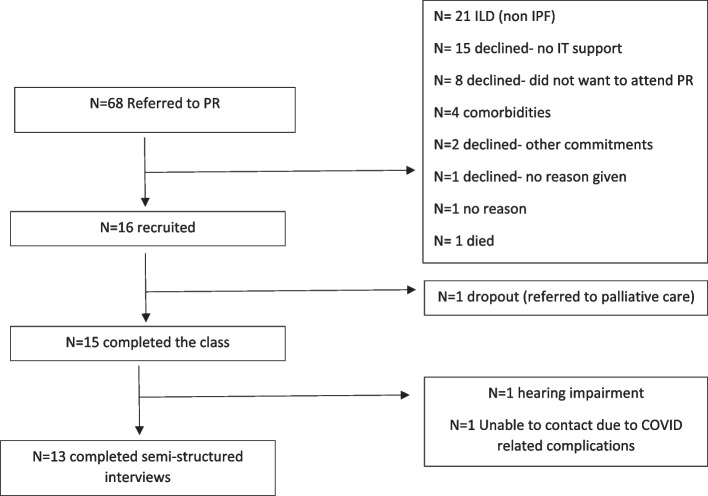
CONSORT Flow diagram

Sixty-eight participants were referred to the programme. Sixteen were recruited, see Fig. 1 for full screening details. Fifteen participants completed the VPR, *n*=1 dropped out as they experienced an acute worsening of their symptoms. Two participants were unable to complete the interviews, *n*=1 due to a hearing impairment and *n*=1 due to COVID-19 related complications. No participants reported having COVID-19 throughout the study period. The hearing impairment did not affect participation in the exercise class as the individual could visually follow the facilitator. We completed 13 interviews. The authors deemed that thematic saturation had been met at 12 interviews, and conducted a further one to confirm no further themes were being identified. The interviews ranged from 12 to 27 minutes in duration. See Table 2 for an overview of participant characteristics. No participants had previously participated in PR.

**Table 2:** Participant demographics

Parameter	Mean (Standard deviation)
Gender	*N*= 7 Male *N*= 6 Female
Age	69.5 (10.4) years
FEV_1_ (Forced expiratory volume in the first second of a forced expiratory manoeuvre)	2.6(0.3)L
FVC (Forced Vital Capacity)	2.9(0.4)L
Attendance	18.5 (1.6)

Based on our analysis, we generated four core themes: (1) The impact of VPR on health and outlook, (2) The reality of VPR, (3) Being active after VPR and (4) Living with IPF during the COVID-19 Pandemic. We now describe these four themes and include representative quotations e.g. P101F 52 yrs, is participant 1 (female) aged, 52 years.

### The impact of VPR on health and outlook

The majority of participants described improvements in their health and wellbeing as a result of participating in the VPR programme. For some these improvements were wide reaching, affecting multiple facets of their lives.

*“No words could describe. It's very overwhelming, the positivity of it. It's the best invention ever. It's done me the world of good anyway. And the other thing is, that I've noticed with my family members, from my children, is they'd come down…they could hear me on Zoom doing the physio. And they enjoyed it and they thought... it was a positive effect for them, and they seen me getting on with it... The smile on their faces and my family, my children. It's been brilliant.”* (101F 52 years)

Others found it “*difficult to explain,”* (105M 78 years) the benefits but found they were “*a bit looser…and in better form…more chatty and more joking or whatever it is*.” (105M 78 years) or described a feeling of renewed hope “*I just gets me motivated a little bit. I haven't had any of that for a long, long time.”* (109F 66 years)

Others described specific improvements in their condition relating to respiratory health:

“*Just the cough. I feel as if I'm actually getting me life back. I've been attending the hospital for the last five years. I've never really got any relief from the cough. I even used to get to the stage where...when I used to wake up in the morning I'd say, "I don't want to do this anymore." because I couldn't stop coughing. But now, when I wake up, in the morning I look forward to the day. I haven't done that for years.”* (109F 66 years)

Despite these profound improvements for some individuals, a small number of participants did not experience improvements, one participant reported being “*no worse off than,”* (112M 73 years) before the programme. For others the rapid deterioration in their functional status as a results of IPF made it difficult for them to observe any improvements.

*“This has crept up on me over the last three years. Two years ago, I could walk a golf course. No problem. Now, I wouldn't walk one hole, I was puffing and panting and having to stop several times for a breath. You know what I'm saying? So I can't, what am I comparing it with? I wouldn't have a clue*.” (108M 82 years)

The progressive nature of the disease means that some felt worse even after a PR programme.

“*I'm probably worse but it's nothing to do with the program. I've not been well since Christmas, I think…I feel, it would definitely would have made me feel better and all that. But since I've gotten worse I can't compare, I wouldn't say it helped me get better*.” (106M 71 years)

Despite the mixed response to changes in health and wellbeing all participants reported that they “just *enjoyed it all*.” (105M 78 years) and had a “*little bit of a banter*” (102M 61 years). This enjoyment was in part due to the facilitator who was described as “*fantastic,”* (101F 52 years), and as having “*a great personality,”* (112M 73 years). One participant remarked that “*You wouldn't anyone find better than Grainne*.” (105M 78 years). Even participants who reported no improvement in their health reported that they would do the programme again if offered.

*“If asked “would you like to try again," I would even though, I mightn't be fit enough to complete all the exercises, but I still would because it was enjoyable.”* (106M 71 years)

### The Reality of VPR

Most participants were satisfied with the duration and frequency of contact of the programme and would recommend it to other individuals with IPF.

*“I think it was a great help. And I think it'd be a great help to anybody else that's in my position, to be honest with you, for people, just to make them better and do these exercises.”* (103F 73 years)

Any suggestions for improvement were mainly that the programme should be “*a little bit longer*” (102M 61 years) or be more frequent, up to “*three times a week,*” (108M 82 years). Some participants, completed the programme during summer, where it was felt it “*might've been clashing with people's holidays and things like tha*t,” and that “*if it was shortened to three days a week or something, might be better*,” (111M 74 years). Despite the varied opinions around the duration and frequency of contact, adherence to the programme was reportedly quite good. Most participants reported that they “*never miss(ed) it*,” (105M 78 years). While a small number of participants missed classes due to holidays, work and other commitments

*“I missed two classes one week, we were away.”* (112M 73 years)

The remote nature of the programme likely enhanced adherence as there was “*no driving and there's no long waits, and it's all done in an hour, or a little over an hour*,” (112M 73 years) furthermore participants didn’t have to worry about “*having to park*” (102M 61 years) as they would have had to do if the programme was delivered in person. It also afforded participants who were still working some flexibility, as participants only, “*had to arrange with the manager to let me take an hour off each day*.” (101F 52 years). Additionally the virtual aspect facilitated participants to work up to and immediately after the VPR session.

“*Well, I was lucky in this sense because my boss allowed me an hour. So I went into a private room and I could do the exercise.”* (102F 52 years)

There were mixed views about the group dynamic in the virtual environment. Some felt that “*there wasn't that much chat between us* (the group),” (106M 71 years) and that they would have benefited from “*a five minute introduction to each other,”* (111M 74 years). However, for others despite exercising alone at home some felt that they weren’t “*totally,”* (104M 75 years) alone or that “*if you tuned in five minutes before you'd have a chat with the other people*,” (107F 73 years). This feeling of being in group was not always a positive, as part of the monitoring participants had to call out their oxygen saturations which could be perceived as demoralising.

“...*there was people 15, 30 years older than me getting their oxygen levels that were 10% higher than me. Now, that was concerning, do you know what I mean*?” (101F 52 years)

Despite being in group environment participants were still afforded the opportunity for one-to-one consultation with the supervisor after class which was valued “*because you weren't kind of broadcasting your fears in front of everybody else, it was just between you and her,”* (102M 61 years). Others questioned “*how people are selected*” (111M 74 years) as “*there was a man on it who was on oxygen and nobody else was on oxygen,”* (111M 74 years) or “*there was a lot older people on the program and some of the exercises were enough for them,”* (109F 66 years). All participants felt safe exercising remotely given the small size of the group and could have one-to-one consultations with the facilitator as required.

“*There was four of us in the class and she could see us all and she was able to know if you were getting too out of breath, or she could tell you what to do, or anyone that was on oxygen to lower or higher the oxygen.”* (107F 73 years)

Finally, there were mixed experiences with the technology, some had, “*no issues,”* (111M 74 years) or “*had a problem at the beginning because my password wasn't right,”* (112M 73 years). Others had issues at times, “*when the Wifi was bad*,” (101F 52 years) but overall participants were quite positive about their experience, even those who had limited technological experience.

*“Now, I'm not tech minded. I can use me tablet to a certain degree. Just certain things. I can look up emails, or news, that kind of thing. I always do that. But when she said to me about Zoom. I had heard of Zoom, I hadn't a clue what it was or... And she talked me through it, and with great patience I have to say, because I was full of questions.”* (107F 73 years)

### Being active after VPR

Being active after VPR

Participants had very mixed responses regarding their engagement with physical activity having finished VPR. Motivation was frequently mentioned, some were motivated by the sense of “*achievement*,” (102M 61 years) of a step goal they had set themselves. Others were motivated by health reasons, highlighting the prospect of a “*transplant*” (113F 47 years) as a motivator or the benefits they had achieved from the VPR.

*“Well, it's just the improvement I see in myself is the main motivation, because I'd given up. I'd said, "No, I'm as I am, and that's it. I'm not going to be any better." Whereas, I was totally wrong. I certainly am better. So I mean, that's the motivation really*.” (104M 75 years)

However not all participants expressed the same level of motivation, as it is more difficult “*when you're left to your own devices,”* (103F 73 years), however they still tried to engage in some activity

“*I'm not giving the same time to it, if you know what I mean. Like 15 or 20 minutes, I have enough. Where when I was with somebody in front of me I was doing the hour.”* (107F 73 years)

Other external barriers to maintenance were highlighted including “*working a 12 hour shift,”* (102M 61 years), comorbidities including “*pain,”* (103F 73 years), or “*weather,”* (103F 73 years), *“time,”* (111M 74 years) and reduced functional status.

*“ I find it very difficult. I used to be able to keep the garden, mow the grass. I can't do any of those now. I have to stand over somebody and tell them what to do and they might not do it the way I want to*.” (106M 71 years)

Facilitators to maintenance included having equipment at home for example “*an exercise bike*,” (104M 75 years), or having “*joined the exercise class*,” (107F 73 years) delivered by a support group and social support. A majority of participants expressed a desire for ongoing access to PR.

“*My daughter is training to be a personal trainer as well at the moment. And I've been doing great exercises with her……we were doing classes online.”* (113F 47 years)

### Living with IPF during the COVID-19 Pandemic

The VPR was delivered during periods of varying levels of restrictions during the COVID-19 pandemic and some participants reported their experience of living with COVID-19. Some participants described being “*very fearful*,” (101F 52 years) and they would be “*a little bit concerned about it when you'd out anywhere or at funerals and that, because you have to stand well back and all that,*” (105M 78 years). The restrictions reduced people’s activity, as they “*haven't really been going out very much [due to restrictions], you can't do a lot*.” (103F 73 years). For, some the VPR gave them a focus away from COVID.

*“It just helped me so much, particularly in the times of COVID. I just was at my wits end. I just didn't want to move. I really and truly wasn't getting any exercise. None. After I'd have my breakfast in the morning I'd come and sit down and really that was me for the day. Unless maybe going out to make a meal or that I was just sitting around and knew there was nobody going to be calling, and oh, it was just horrible.”* (107F 73 years)

## Discussion

This study successfully achieved its aim of exploring people with IPFs experience of VPR. There were a number of key findings in the current research. Participants in the current study were very satisfied with the VPR programme. Participants reported a number of physical and psychological benefits and there was high praise for the facilitator. The remote delivery of the programme enhanced accessibility, participants felt safe exercising in this environment and any issues with technology were easily overcome. There were calls for the increased duration and frequency of delivery of the programme. The timing of this research during the COVID-19 pandemic provided a unique opportunity to explore the views and experience of this vulnerable population; the VPR provided an outlet for exercise for people with IPF who were otherwise advised to cocoon at home.

COVID-19 saw a rapid shift to the provision of remote health services including PR [17]. A recent systematic review reported that remote PR for people with CRD achieves outcomes similar to those of traditional centre-based pulmonary rehabilitation, with no safety issues identified [18]. Furthermore, tailoring of PR programmes including the provision of remote PR on a routine basis is advocated [17, 29]. Participants in the current study regardless of whether they experienced health benefits as a result of participating in the programme reported high levels of satisfaction with the programme. Participants were particularly satisfied with the facilitator of the VPR, the views and experience of the facilitator were not captured in this study. Future research should consider exploring the views of those facilitating VPR programmes in addition to what qualities are important in a facilitator to promote satisfaction among participants. A small number of participants reported issues with the technology but these were easily overcome. There is some evidence of increased dropout in VPR among older more frail adults [30], however we observed only one dropout secondary to acute worsening of their symptoms. Ease of accessibility to the programme was highlighted by participants which is in line with current research [31]. PR is conducted in a group setting and for some this was not lost in the virtual environment, participants still felt as though they were connecting with others. However, some participants felt that they missed this social aspect and time could be set aside for group interaction although conversely the social support provided by family members while exercising in the home was noted by some. Some participants also reported being uncomfortable with reading their oxygen saturations to the group, VPR provision should allow for the remote transmission of participants vitals to the facilitator, for which the current study did not have the resources for. Future researchers and practitioners should explore means of facilitating group interaction in remote PR. Finally it must be noted that a number of potential participants declined the programme due to the remote nature, reasons for this were not explored. Potential reasons include a lack of confidence with technology which could be overcome with more formal training or “zoom fatigue,” [32] as several services moved to online platforms including not only medical but educational, religious and recreational services. This further strengthening the calls for the provision of choice for people referred to PR and/or additional training with technology for individuals.

While PR is recommended for people with IPF at a national and international level [10, 11, 33]; there are no specific guidelines for PR in this population. Current PR guidelines from the Irish Health Service recommend a programme lasting a minimum of 6 weeks [10], while the British Thoracic Society recommend a programme of 6-12 weeks [34]. However the current evidence to support the role of PR for people with IPF mostly includes programmes of 8-12 weeks in duration [12]. Furthermore current evidence indicates that a minimum of 16 weeks is advised in healthy older adults in order to improve aerobic fitness [35]. Participants in the current study mostly reported they would prefer a programme of increased duration. Additional work is required to determine the optimum duration of the programme, taking into account a cost benefit analysis. The ratio of participants to facilitator in the current study was 1:4 which was valued by participants. The current recommended ratio for PR in the current guidelines is between 1:4-6 [11, 34], it is not clear whether this should be adjusted for remote PR. The pathophysiology of IPF can result in rapid oxygen desaturation on exertion [12] which may require closer monitoring than other CRDs. A recent study exploring the provision of PR in Ireland, found that facilitators who cater for multiple CRDs adapt their programme for people with IPF with closer monitoring of oxygen saturations and providing supplemental oxygen [36]. Additional research is required to determine optimum ratios for PR for people with IPF. Finally the range in participant clinical presentation and age was noted by some participants which caused them to question their suitability for the programme. Clinicians should provide potential participants with sufficient information about the programme to prepare them for these individual variations. It should also be highlighted at this time that current evidence indicates that people with IPF have greater sustained improvements in functional exercise capacity when PR is delivered early in the course of disease [29].

Maintenance of the gains made from participation in PR has proved challenging [37]. Dowman et al. 2021 reported that, people with ILD maintained benefits in exercise capacity, quality of life and dyspnoea six to 12 months after PR, however these sustained improvements were less certain for people with IPF [12]. This is unsurprising given the progressive nature of IPF. We explored participants’ confidence for continued engagement in physical activity following PR. Participants’ expressed a number barriers and enablers for maintenance. Barriers included work, weather, pain, time and functional status as well as a need for an external motivator. Enablers included social support, access to facilities and internal motivation. These barriers and enablers are not unique to people with IPF and have been previously documented [38–40]. There is an argument that a behaviour change intervention following PR could promote sustained engagement in physical activity and consequently in the gains attained during PR [29]. An enabler for engagement in physical activity more unique to IPF and other CRDs is was the prospect of a lung transplant. There is evidence for the importance of maintaining functional capacity and preventing further physical deterioration in those awaiting lung transplantation [41]; as exercise capacity is an important predictor of mortality [42, 43] and post-transplant survival [44, 45]. There is therefore a need to explore the feasibility of a physical activity behaviour change intervention for people with IPF focusing on their individual barriers and enablers for maintenance.

People with pre-existing interstitial lung diseases including IPF are reported to have a higher incidence of COVID-19 and higher severity and morality should they become infected [46]. As such people with IPF were identified as a vulnerable group and advised to cocoon by the Irish government [16]. The Irish Lung Fibrosis Association (ILFA) conducted a survey to gain an understanding of the impact of COVID-19 on the daily life, healthcare needs, well-being and outlook of people with ILD and their care givers [47]. This survey found that participants with ILD reported a higher level of worry with regard to COVID-19 than their healthy counterparts [47]. Participants in the current study described the fear surrounding COVID-19. Interestingly the survey conducted by ILFA found that two thirds of participants were not confident in the health services ability to meet their healthcare needs [47]. The regular access to a healthcare professional during the PR programme was valued by participants in the current study and was possibly exaggerated due to the timing in relation to COVID-19. The survey also reported that emotional distress and the negative impacts on daily life were more common in caregivers than the patients [47]. Significantly the positive impact of PR on the wider family was noted as the participants could be observed engaging in PR at home. The full impact of COVID-19 on people with IPF is not yet fully understood nor was it our aim to explore this. There does however seem to be some evidence that participants in PR during the COVID-19 pandemic and associated lockdowns helped to mitigate some of the negative consequence on people with IPF.

This novel research reports on people with IPF experience of VPR during the COVID-19 pandemic. There was an overwhelming positive response to the VPR, which is noteworthy given the chronic progressive nature of IPF and the associated life expectancy; against all of the odds the VPR was able to make these individuals feel better. VPR appears to be feasible for people with IPF, with high adherence rates and no adverse events as demonstrated in the current study. We believe the findings of this research are translatable to other centres given the facilitator is experienced in delivering PR and participants have access to the technology. This research is not without its limitations. While data saturation was met, given the limited body of research available in this field and the low recruitment rate it is not clear how representative this data is. Future research would benefit from purposive sampling to ensure representation across factors such as age, disease status, social status (e.g. living alone, occupation) and whether patients are awaiting a transplant. Furthermore the current VPR programme was specifically for people with ILD, the experience of people with IPF attending a mixed PR programme may differ. Patients under the palliative care team were not included in the current programme and one interview was not conducted as the participant had a hearing impairment. Future research should explore the views of those undergoing palliative care and provide a means of conducting interviews for those with hearing impairments or other language difficulties.

In conclusion VPR is an enjoyable experience for people with IPF. The results of this research provide valuable learning on the impact of COVID-19 on the people with IPF and PR services. Most participants in the current study reported benefits to participation. VPR allowed for enhanced accessibility and may provide a viable sustained alternative to centre-based PR. Future work should explore maintenance post VPR and the optimum duration of VPR.

## Supplementary Information

**Additional file 1.**

## Abbreviations

IPF: Idiopathic Pulmonary Fibrosis
Pulmonary Rehabilitation: Pulmonary rehabilitation
VPR: Virtual Pulmonary Rehabilitation
OOS: Orlagh O’Shea
GM: Grainne Murphy
KOR: Katherine O’Reilly
LF: Luke Forde
CRD: Chronic Respiratory Disease
