# Supplementary material for: A qualitative exploration of people living with idiopathic pulmonary fibrosis experience of a virtual pulmonary rehabilitation programme
Source: BMC Pulm Med. 2022 Nov 28;22:448. doi: 10.1186/s12890-022-02221-6 (PMC9702935; doi:10.1186/s12890-022-02221-6)
Supplement: Supplementary file 1 — Additional file 1. [file 12890_2022_2221_MOESM1_ESM.docx]

E supplement

The intervention

The programme was delivered through a platform called Salaso. This platform allows us to do individual and group video calls and also allows us to send questionnaires and exercise programmes. Once a participant had agreed to participate in the VPR they were sent an oxygen saturation monitor a paper copy of the BORG scale by post. A virtual assessment was then conducted including an assessment of the environment for safety as per the facilitator’s clinical judgement.

Participants are introduced to each other in the first class. There was a maximum of six participants per class and participants could interact with each other and the facilitator. Participants participated in a series of exercises including a warm up, upper and lower limb strengthening exercises (e.g. squats, shoulder press) and aerobic exercises (e.g. marching on the spot, heel taps). Participants progressed to using weights, which were obtained by participants themselves. Oxygen saturations, heart rate and BORG are documented at the beginning of the class and intermittently throughout the class. See images 1 and 2 for visual representation of the facilitator delivering the programme.

Two formal education sessions were delivered: the benefits of exercise (including a home exercise programme) and Conservation of Energy. Breathing control methods were taught throughout each class and relaxation was performed at the end of each class. Individual consultations with the facilitator were facilitated on an informal basis before or after the class at the request of participants.

Image 1 Image 2


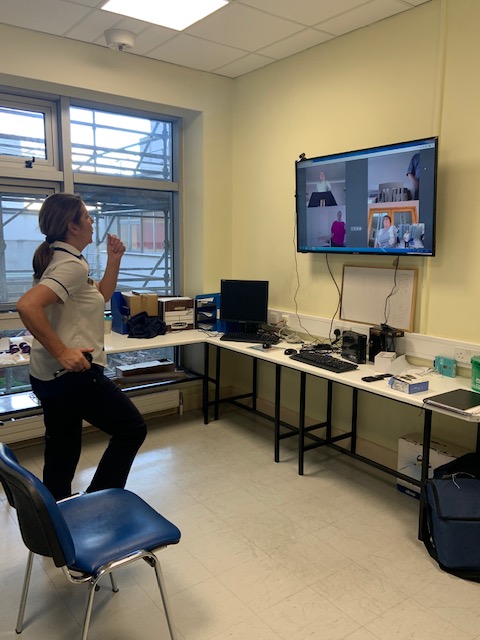

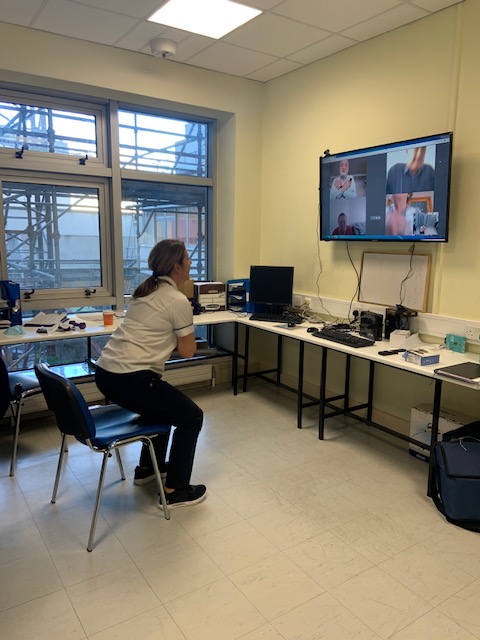


COVID-19 restrictions Ireland

Restrictions varied throughout the study period from level 5 (October to December 2020 and December 2020- May 2021). Level 5 restrictions included for example no social gatherings, travel restricted to 5km from home except for work, people were advised to work from home where possible and all non-essential retail was closed (<https://www.gov.ie/en/press-release/066ce-ireland-placed-on-full-level-5-restrictions-of-the-plan-for-living-with-covid-19/>). There was a gradual reopening of society for example non-essential retail could initially provide click and collect and services by appointment, in May 2021 and restaurants and bars could provide outdoor dining in June 2021. The full timeline of restrictions can be viewed at <https://www.youtube.com/watch?v=BF6oLSwKMF0>

Stages of Braun and Clarke Analysis

The steps involved in the framework are listed as follows:

1. Step one includes familiarising yourself with data through multiple readings.

2. Step two generates an initial list of ideas about what is in the data and what is interesting about them and involves the production of initial codes from the data.

3. Step three, themes begin to emerge, and this refocuses the analysis at the broader level of themes.

4. Step four involves reviewing themes whereby a set of candidate themes are explored and refined, including similarities and differences between interviews.

5. Step five involves defining and naming themes
